# Knowledge abstraction and filtering based federated learning over heterogeneous data views in healthcare

**DOI:** 10.1038/s41746-024-01272-9

**Published:** 2024-10-16

**Authors:** Anshul Thakur, Soheila Molaei, Pafue Christy Nganjimi, Fenglin Liu, Andrew Soltan, Patrick Schwab, Kim Branson, David A. Clifton

**Affiliations:** 1 Department of Engineering Science, University of Oxford, Oxford, UK; 2 Oxford University Hospitals NHS Foundation Trust, Oxford, UK; 3 GlaxoSmithKline, London, UK; 4 Oxford-Suzhou Centre for Advanced Research, Suzhou, China

**Keywords:** Health care, Biomedical engineering

## Abstract

Robust data privacy regulations hinder the exchange of healthcare data among institutions, crucial for global insights and developing generalised clinical models. Federated learning (FL) is ideal for training global models using datasets from different institutions without compromising privacy. However, disparities in electronic healthcare records (EHRs) lead to inconsistencies in ML-ready data views, making FL challenging without extensive preprocessing and information loss. These differences arise from variations in services, care standards, and record-keeping practices. This paper addresses data view heterogeneity by introducing a knowledge abstraction and filtering-based FL framework that allows FL over heterogeneous data views without manual alignment or information loss. The knowledge abstraction and filtering mechanism maps raw input representations to a unified, semantically rich shared space for effective global model training. Experiments on three healthcare datasets demonstrate the framework’s effectiveness in overcoming data view heterogeneity and facilitating information sharing in a federated setup.

## Introduction

The healthcare landscape is being revolutionised by machine learning (ML) advancements and the shift towards patient-centred care, with Electronic Health Records (EHRs) providing critical medical data essential for transforming healthcare delivery^1^. However, privacy regulations such as the General Data Protection Regulation (GDPR)^2^ and the Data Protection Act (DPA)^3^ restrict EHR sharing, causing datasets to be distributed among institutions while limiting ML models to specific populations, thereby hindering their generalisation^4^. Federated Learning (FL) offers a solution for this challenge by enabling collaborative training of ML models using data distributed across multiple institutions^4–6^. FL follows a classical client-server architecture where institutions act as clients, and a central server coordinates the training of a global model. In each iteration, the server sends the current global model to the clients, who update it using their local data and send the updated models back to the server. The server then aggregates these client-specific models to form an updated global model, a method known as Federated Averaging (FedAvg)^7^. Another variant, federated stochastic gradient descent (FedSGD)^5^ or distributed synchronous SGD^8^, involves clients computing and sending gradient updates rather than the updated model to the server, which then aggregates these gradients to update the global model. As a result, FL ensures data privacy by eliminating the need for direct “outside” access to the data stored within institutions or clients.

**Fig. 1:**
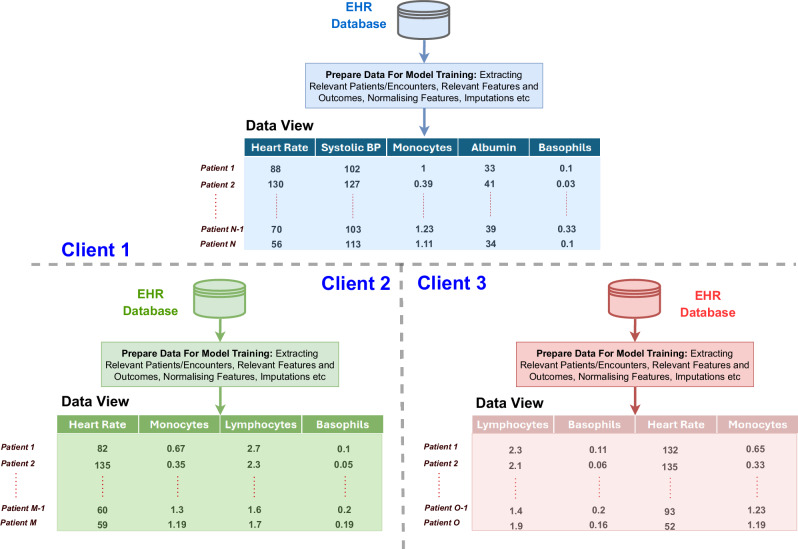
Illustration of data view heterogeneity in ML-ready data views. A hypothetical scenario illustrating data view heterogeneity across three clients in a federated learning setup. Client 1 has different features compared to Clients 2 and 3, while Clients 2 and 3 have shuffled data views. This scenario requires additional processing over the client-server communication channels to align the data views, such as selecting a common subset of features for each client and ensuring consistent feature locations across all three data views.

The distributive and privacy-preserving nature of FL holds immense potential for improving healthcare informatics. However, the nature of existing FL solutions in non-imaging and non-text clinical applications necessitates consistent structures of training data across clients. The structure of training data, referred to as the ML-ready *data view*, can vary among clients, leading to what we call *data view heterogeneity*. Each client independently processes their respective Electronic Health Record (EHR) databases to obtain these ML-ready data views. Common sources of heterogeneity in data views across clients include feature disparity (i.e., differences in the number of features), shuffled data views (i.e., variations in the location of features within feature vectors), and differences in scales or units of features. Figure 1 illustrates a common case of data view heterogeneity. Since FL involves collaborative training of a global model, clients exhibiting feature disparity cannot structurally process their local data using the global model. For example, a global model expecting a *d*-dimensional input cannot process a client’s data view with *m*-dimensional feature vectors. Additionally, the presence of shuffled data views and differences in feature scales across clients results in updated local models that are inconsistent with each other, thereby hampering the training of the global model. Therefore, data view heterogeneity presents a significant challenge in employing FL effectively.

In the clinical context, data view heterogeneity is especially pronounced. Every medical institution collects and stores data differently, with variations in available medical services and standards of care further compounding disparities in EHR datasets. Inter-hospital differences include diverse methods for collecting and recording clinical results, as well as the use of distinct assays. This leads to heterogeneity in units, reference ranges, and result interpretation. These disparities are even more significant in low- and middle-income countries (LMICs), where medical service availability and care standards vary dramatically by region^9,10^. Each institution, acting as a client, processes its EHR database to prepare ML-ready data (Fig. 1). As a result, these inconsistencies in EHR datasets carry over to the data views, creating heterogeneity across clients and preventing direct FL application. To enable FL in such cases, labour-intensive preprocessing is required, such as selecting a subset of common features, standardising measurement units, and reordering features across institutions. The nature of this preprocessing task presents the following challenges:

- The distributed nature of data view alignments imposes an additional burden on client-server communication channels in FL setups. Since clients cannot communicate with each other in a FL setup, the communication needs to go through the server.
- Apart from preprocessing EHR databases to prepare data views, additional manual effort is required at each client to also facilitate the preprocessing operations to align data views, which may discourage institutions from participating in federated training.
- The omission of crucial features at some institutions to align data views risks losing invaluable insights. Additionally, omitted features cannot be used in the personalised global model^11^, a global model fine-tuned by a client for its population. This omission could further diminish the benefits of participating in FL for some clients, as they won’t be able to fully utilise the available features that might be essential to their populations for certain prediction tasks.

Despite these challenges, manual alignment has been employed in earlier studies to facilitate FL. For instance, in our previous CURIAL study^12^, we trained a global federated model for COVID-19 prediction using vital signs and blood panel data distributed across four NHS trusts. To ensure data consistency, we harmonised data views by selecting a common subset of features, which was crucial due to the unavailability of specific features for certain cohorts at particular times. However, the omission of certain features in some trusts diminished the model’s potential performance. On a different note, common data models (CDMs) offer a potential solution to address inconsistencies in the structures of distributed EHR data and, consequently, in ML-ready data views^13,14^. While CDMs aim to standardise formats, they may inadvertently lead to information loss^15^, hindering the development of more robust models. Additionally, the adoption of CDMs remains limited, with only 12% of EHR systems supporting them as of 2022^16^.

While few studies directly address federated training over heterogeneous data views, many have focused on data or statistical heterogeneity^17^. Statistical heterogeneity arises from distribution shifts in clients’ local data, leading to conflicting gradients or model updates that hinder global training^18^. Data view heterogeneity, where clients have shuffled feature sets, can be seen as an extreme form of statistical heterogeneity. In addition to statistical challenges, model heterogeneity-where clients use different model architectures-has also been explored^17^. These studies typically align global and client model outputs using shared or generated samples^18,19^. However, this requires all models to process the same examples, which is problematic when clients have varying input dimensions. To address this, approaches like Local Global Federated Averaging (LG-FedAvg)^20^ train only the classification layers on the server, while feature extractors remain local. This allows clients to project local data into a shared dimensional space for global classification. Such methods, similar to multi-source domain adaptation, effectively manage feature heterogeneity by projecting data from different sources into a fixed-dimensional space^21,22^. However, relying on local feature extractors limits information sharing among clients and can induce distribution shifts, as each extractor is trained independently.

In addition to these studies, a few frameworks have directly tackled data view heterogeneity. Hypernetwork-based FL^23^ provides a theoretical solution by training a global hypernetwork at the server, which generates weights for personalised client models based on each client’s unique data view. While promising, hypernetworks are difficult to train, offer limited information sharing between clients, and require substantial architectural changes if the target model changes. Building on this, we recently proposed an Augmented Graph Attention Network (AGAT)-based FL framework^24^, which uses data augmentation and GAT’s ability to prioritise relevant features to address feature disparity among clients. Although effective, this method may face challenges when dealing with significant feature differences and shuffled data views. Hence, there is still a requirement of an effective FL solution that can operate on heterogeneous data views without requiring any manual preprocessing and data alignment.

**Fig. 2:**
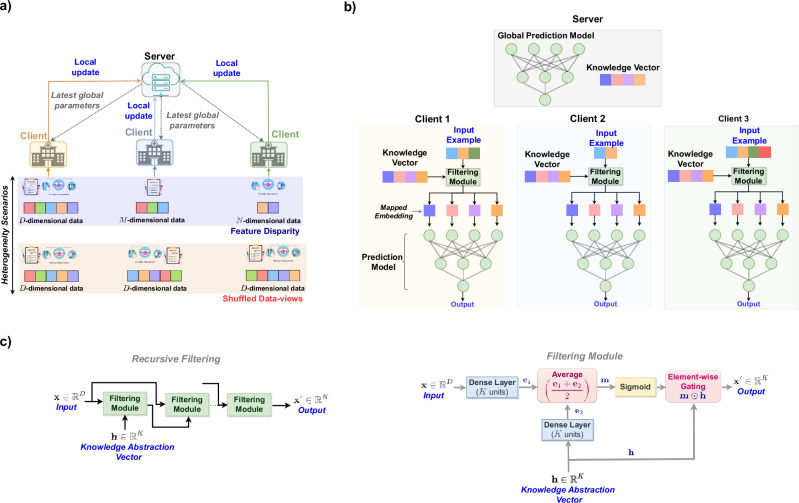
Illustration of data view heterogeneity in Federated Learning (FL) setups and the components of the proposed framework designed to manage this heterogeneity. **a** An illustration of data view heterogeneity arising from feature disparity and shuffled features across 3 clients in a federated learning framework. **b** A systematic illustration of the proposed knowledge abstraction and filtering mechanism, enabling a global model with a predefined architecture to process diverse data views across clients. The predictive model expects a 4-dimensional input, matching the dimensions of the knowledge vector. At each client, the filtering module takes in the knowledge vector provided by the server and an input example, regardless of its dimensions, and outputs a 4-dimensional filtered version of the knowledge vector, which is then fed to the prediction model. **c** Block diagrams illustrating the architecture of the proposed filtering module and the process of recursive filtering implemented with it.

**Fig. 3:**
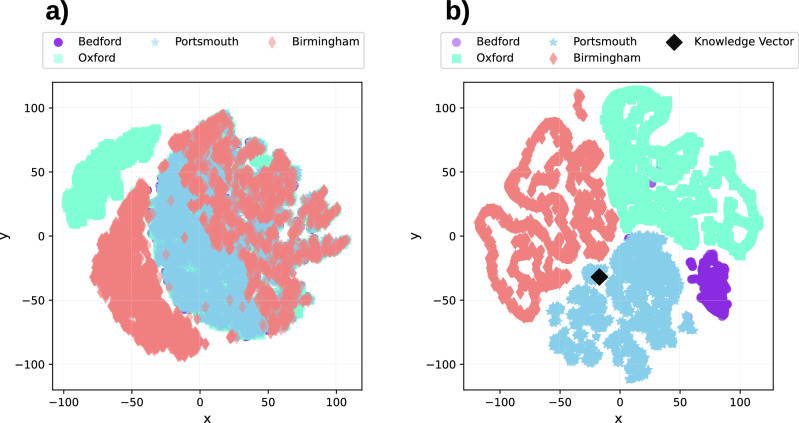
Nature of shared representation space learned by the proposed framework. 2-d t-SNE^25^ representation is illustrated for both: (**a**) the input data space and (**b**) the shared representation space obtained after knowledge filtering, for each client in the CURIAL dataset.

In this paper, we propose such a solution, offering a novel FL framework capable of addressing data view heterogeneity efficiently (Fig. 2). Central to this framework is a transformative *knowledge abstraction and filtering* mechanism (Fig. 2c), which maps raw input representations into a unified, semantically rich shared space across clients, facilitating consistent training over heterogeneous data views. This mechanism hinges on a global trainable vector, referred to as the knowledge vector, which amalgamates information from all clients in a latent manner. Each client then employs client-specific filtering modules (not trained using FL) to extract relevant information from the knowledge vector, projecting input examples into the unified shared space. The resulting mapped embeddings then serve as input to the prediction models, thereby ensuring that these models are trained on a consistent space. Figure 3 illustrates the nature of this shared space learned for the four CURIAL clients using 2-d t-SNE^25^ representation. Importantly, this knowledge abstraction and filtering mechanism serves as the first module of a task-specific model, making it compatible with various neural network architectures and enabling seamless integration with any FL framework. This “plug-and-play” capability enhances the versatility and applicability of the proposed approach.

## Results

### Datasets

The efficacy of the proposed framework is demonstrated on the following datasets:

- **CURIAL:** The CURIAL datasets^12,26,27^ consist of anonymized electronic health record (EHR) data (including demographic information, blood tests, and vital signs) from emergency departments across four independent United Kingdom (UK) National Health Service (NHS) Trusts: Bedford, Oxford, Portsmouth and Birmingham. Each Trust is considered as an independent federated learning client. These datasets are used for the binary classification task of diagnosing COVID-19.
- **eICU Collaborative Research Database (eICU-CRD):** The eICU^28,29^ is a large multi-centre dataset containing 164, 333 patients’ ICU stays as time-series samples, collected from 150 different hospitals. In this study, each hospital is considered as separate federated client having its own dataset. To ensure effective training and evaluation, we have included only the 50 hospitals with the highest number of ICU stays, totalling 107, 723 stays. For each hospital, we use a pre-processed version^29^ of this dataset for predicting *shock* in the next 4 hours. Each ICU stay is represented by a time-series containing 4 time-steps where each time-step is represented by the 254-dimensional feature (discussed in Supplementary Note 1).
- **MIMIC-III**^30,31^: This dataset contains extensive patient information from a critical care unit, pre-processed to represent each ICU stay as an evenly spaced time-series where each time-step is represented by 60 clinical features. We use this dataset to perform *mortality prediction* based on the first 48 hours of ICU stay and *phenotyping* the ICU stays into 25 categories. To simulate federated setup, we employ random splits to divide the available examples into ten distinct sub-datasets, and each of these sub-datasets is then assigned to a simulated client.

Table 1 provides a summary of these datasets, including details such as the number of examples and simulated clients. In all datasets, 65%, 15%, and 20% of the available examples at each client are designated for training, validation, and testing, respectively. Additional information about the available features in each dataset, along with the number of examples at each client, is provided in Supplementary Note 1.

**Table 1:** Details of the datasets used for the performance evaluation

Datasets	# Examples	Nature	Task	Clients
CURIAL	297,773	Tabular	COVID-19 Prediction	4
eICU	107,723	Time-series	Shock Prediction	50
MIMIC-III	21,139	Time-series	Mortality Prediction	10
MIMIC-III	41,902	Time-series	Phenotyping	10

### Baselines and evaluation scenarios

The performance of the proposed framework is compared against the standalone models (models trained at each client), standard federated averaging (*FedAvg*)^7^, hypernet-based FL^23^, local global federated averaging (LG-FedAvg)^20^ and augmented graph attention networks (AGAT)^24^. All methods are evaluated in the standard FL setting as well as in presence of heterogeneous data views. The evaluation scenarios are listed below:

- STANDARD: This is a classical FL scenario where all clients have identical data views. For datasets with inherent data view heterogeneity, we selected a common subset of features across all clients.
- FEATURE DISPARITY: In this scenario, the number of features varies among different clients. We simulate this scenario by random changing the number of available features at different clients. For example, two CURIAL Trusts possess 21 unique clinical features, while the remaining Trusts contain 28 features in their respective datasets. The details about the dimensionality of data at each client is provided in the supplementary document (Supplementary Note 1). Since *FedAvg* cannot be implemented in this scenario, we manually harmonised the data views by selecting the common subset of features across clients. This results in loss of features at all but one client.
- SHUFFLED DATA-VIEWS: In this scenario, features across different clients are same. However, different features occupy different location on the feature vector. We simulate this scenario by randomly shuffling the data views at different clients.
- DIFFERENT MEASUREMENT UNITS: This scenario underscores the significant variation in measurement units among different clients. For example, one client might have their Bilirubin levels measured in milligrams per decilitre, while others use micromoles per litre. We simulate this scenario by artificially changing the units/scale of clinical features in the CURIAL dataset across clients. MIMIC-III and eICU mostly contains binarised features and hence, cannot be used here.

The average area under the ROC curve (AUROC), average area under the precision-recall curve (AUPRC), sensitivity, specificity, positive predictive value (PPV) and negative predictive value (NPV) across all clients are used as the performance metrics to compare baselines. Each client possesses a set of test examples, and the performance of all federated baselines on these test examples is evaluated after local training (or personalisation) of the global model at each client.

### Performance on the CURIAL datasets

**Fig. 4:**
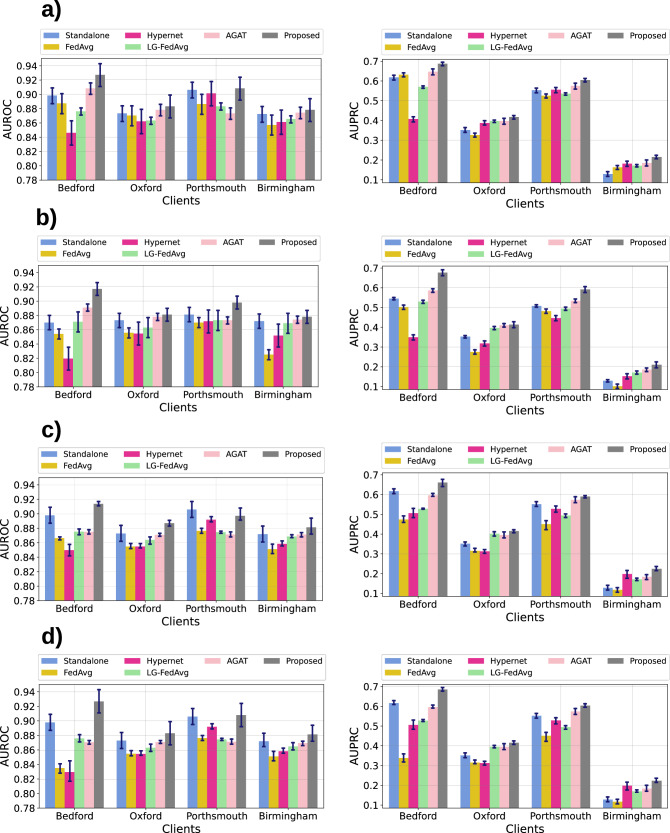
Performance evaluation on the CURIAL datasets. Performance of the proposed framework on the CURIAL datasets in (**a**) standard, (**b**) feature disparity, (**c**) shuffled data views, and (**d**) scaled scenarios. The left column presents the AUROC scores for each individual client, while the right column shows the AUPRC scores.

The performance of the proposed framework and the other baselines on the CURIAL datasets is documented in Table 2 and Fig. 4. The analysis of this figure and table highlights the following:

- The proposed framework demonstrates consistent performance across various scenarios, indicating that data view heterogeneity among clients has minimal impact on its effectiveness. This highlights robustness and versatility of the proposed framework in managing diverse client data. Furthermore, the framework consistently outperforms standalone deep learning models (non-FL baseline) across all scenarios, showcasing its ability to facilitate information sharing among clients despite data view heterogeneity. This information sharing is evident at *Bedford*, a CURIAL site with fewer training samples (Table S2 of Supplementary Note 1), witnesses noticeable performance improvement (average 2.2% in AUROC) over the standalone models in all scenarios.
- In the STANDARD and FEATURE DISPARITY scenarios, AGAT is the best-performing method after the proposed framework, underscoring the importance of graph structures in modelling and the effectiveness of GATs with data augmentation in overcoming feature disparity. Although AGAT outperforms Hypernet and LG-FedAvg in case of shuffled and scaled data views, it does not surpass the standalone models, highlighting its limitations in managing complex heterogeneity.
- In STANDARD scenario, all FL baselines except Hypernet-based FL exhibits comparable performance in terms of both AUROC and AUPRC. As we deviate from the STANDARD scenario, FedAvg experiences a noticeable performance drop. This behaviour is expected due to the presence of feature disparity, shuffled data views, and varying scales at the clients. These factors result in divergent or conflicting gradients or models at the client level, leading to a less effective global model. On the other hand, Hypernet-based FL and LG-FedAvg exhibit no significant deviation in performance based on the presence and nature of data view heterogeneity. However, these methods achieved lesser performance than the standalone models highlighting the weaker information sharing among clients. Since LG-FedAvg only trains the classification layer at the server using FedAvg (with other layers being client-specific), the information sharing across clients is potentially lesser than FedAvg and the proposed framework.

**Table 2:** Average performance of the proposed framework and baselines on the CURIAL datasets acting as clients in a federated learning setup

Scenario	Metrics	Methods					
		Standalone DNN	FedAvg	Hypernet	LG-FedAvg	AGAT	Proposed
Standard	AUROC	**0.874** ± **0.018**	0.88 ± 0.017	0.868 ± 0.021	0.879 ± 0.009	0.883 ± 0.016	**0.897** ± **0.189**
	AUPRC	0.412 ± 0.199	0.431 ± 0.189	0.382 ± 0.139	0.429 ± 0.163	0.45 ± 0.1865	**0.482** ± **0.009**
	Sensitivity	0.851 ± 0.047	0.838 ± 0.065	0.848 ± 0.031	0.841 ± 0.032	0.851 ± 0.011	**0.854** ± **0.008**
	Specificity	0.768 ± 0.038	0.718 ± 0.066	0.684 ± 0.09	0.705 ± 0.057	0.769 ± 0.063	**0.771** ± **0.061**
	PPV	0.147 ± 0.135	0.117 ± 0.094	0.119 ± 0.136	0.121 ± 0.132	0.152 ± 0.148	**0.165** ± **0.162**
	NPV	**0.993** ± **0.005**	0.992 ± 0.005	0.991 ± 0.004	0.991 ± 0.003	0.992 ± 0.004	0.992 ± 0.003
Feature Disparity	AUROC	0.875 ± 0.010	0.852 ± 0.007	0.861 ± 0.016	0.869 ± 0.014	0.879 ± 0.005	**0.894** ± **0.009**
	AUPRC	0.383 ± 0.171	0.332 ± 0.169	0.378 ± 0.111	0.398 ± 0.146	0.429 ± 0.162	**0.472** ± **0.187**
	Sensitivity	**0.853** ± **0.05**	0.846 ± 0.032	**0.853** ± **0.041**	0.852 ± 0.051	0.846 ± 0.032	0.849 ± 0.012
	Specificity	0.746 ± 0.08	0.708 ± 0.07	0.679 ± 0.052	0.752 ± 0.071	0.759 ± 0.062	**0.775** ± **0.05**
	PPV	**0.151** ± **0.101**	0.12 ± 0.113	0.126 ± 0.008	0.131 ± 0.089	0.136 ± 0.122	0.147 ± 0.136
	NPV	**0.992** ± **0.004**	**0.992** ± **0.004**	0.991 ± 0.005	**0.992** ± **0.003**	0.991 ± 0.005	**0.992** ± **0.003**
Shuffled	AUROC	0.878 ± 0.016	0.855 ± 0.011	0.858 ± 0.02	0.869 ± 0.02	0.87 ± 0.02	**0.899** ± **0.024**
	AUPRC	0.412 ± 0.199	0.306 ± 0.126	0.374 ± 0.13	0.397 ± 0.146	0.392 ± 0.17	**0.482** ± **0.186**
	Sensitivity	0.851 ± 0.047	0.831 ± 0.015	0.844 ± 0.006	0.842 ± 0.013	0.847 ± 0.005	**0.852** ± **0.014**
	Specificity	0.768 ± 0.038	0.672 ± 0.036	0.679 ± 0.022	0.698 ± 0.047	0.712 ± 0.033	**0.769** ± **0.038**
	PPV	0.141 ± 0.135	0.105 ± 0.141	0.115 ± 0.087	0.131 ± 0.124	0.133 ± 0.009	**0.161** ± **0.138**
	NPV	**0.993** ± **0.005**	0.986 ± 0.003	0.99 ± 0.004	0.992 ± 0.005	0.991 ± 0.004	**0.993** ± **0.003**
Scaled	AUROC	0.878 ± 0.016	0.862 ± 0.011	0.864 ± 0.017	0.871 ± 0.006	0.872 ± 0.003	**0.892** ± **0.013**
	AUPRC	0.412 ± 0.199	0.34 ± 0.149	0.387 ± 0.144	0.4 ± 0.146	0.433 ± 0.173	**0.474** ± **0.177**
	Sensitivity	**0.851** ± **0.037**	0.838 ± 0.054	0.848 ± 0.044	0.849 ± 0.031	0.847 ± 0.061	0.849 ± 0.011
	Specificity	0.768 ± 0.038	0.685 ± 0.017	0.689 ± 0.037	0.726 ± 0.045	0.706 ± 0.034	**0.773** ± **0.036**
	PPV	0.141 ± 0.135	0.112 ± 0.139	0.121 ± 0.098	0.138 ± 0.132	0.120 ± 0.128	**0.159** ± **0.14**
	NPV	**0.993** ± **0.004**	0.990 ± 0.003	0.992 ± 0.003	0.992 ± 0.003	0.990 ± 0.002	**0.993** ± **0.002**

The performance is measured as the mean across ten independent runs with different initialisations.

### Performance on eICU & MIMIC-III datasets

Table 3 illustrates the average performance of all methods, aggregated from the 50 simulated clients within the eICU dataset. The performance trends observed in the eICU dataset closely mirror those in the CURIAL datasets. Our proposed framework consistently either outperforms or demonstrates comparable performance to all baseline methods across all experimental settings. In the STANDARD scenario, all FL methods exhibit an improvement in average performance over the standalone models. Specifically, FedAvg, AGAT, and the proposed framework achieve comparable performance. Moving to the FEATURE DISPARITY scenario, all methods, including the standalone models, show a significant performance loss due to the availability of fewer features at some clients. Despite this loss, LG-FedAvg, AGAT, and the proposed framework achieve a noticeable improvement, with the proposed framework being the most prominent, over the standalone models. Again, while AGAT exhibits better performance on both standard and feature disparity scenarios, it struggles in dealing with shuffled data views. Meanwhile, LG-FedAvg and the proposed framework achieve better performance than the standalone models. However, the performance boost achieved by the proposed framework is significant.

**Table 3:** Average performance of the proposed framework and baselines on the eICU dataset

Scenario	Metrics	Methods					
		Standalone DNN	FedAvg	Hypernet	LG-FedAvg	AGAT	Proposed
Standard	AUROC	0.681 ± 0.11	0.705 ± 0.105	0.692 ± 0.098	0.698 ± 0.121	0.708 ± 0.116	**0.711** ± **0.091**
	AUPRC	0.224 ± 0.139	**0.257** ± **0.121**	0.229 ± 0.091	0.237 ± 0.121	0.249 ± 0.106	**0.257** ± **0.089**
	Sensitivity	0.516 ± 0.151	0.595 ± 0.168	0.562 ± 0.131	0.57 ± 0.128	0.598 ± 0.107	**0.616** ± **0.161**
	Specificity	0.709 ± 0.069	0.716 ± 0.082	0.684 ± 0.09	0.705 ± 0.057	0.719 ± 0.063	**0.721** ± **0.079**
	PPV	0.143 ± 0.08	**0.168** ± **0.106**	0.139 ± 0.136	0.143 ± 0.132	0.152 ± 0.148	0.167 ± 0.093
	NPV	0.947 ± 0.024	0.955 ± 0.022	0.949 ± 0.032	0.951 ± 0.031	0.949 ± 0.003	**0.956** ± **0.024**
Feature Disparity	AUROC	0.619 ± 0.085	0.602 ± 0.079	0.621 ± 0.013	0.631 ± 0.013	0.637 ± 0.112	**0.658** ± **0.093**
	AUPRC	0.171 ± 0.073	0.155 ± 0.061	0.167 ± 0.132	0.176 ± 0.076	0.183 ± 0.092	**0.203** ± **0.111**
	Sensitivity	**0.563** ± **0.202**	0.514 ± 0.205	0.551 ± 0.041	0.535 ± 0.091	0.532 ± 0.169	0.556 ± 0.184
	Specificity	0.608 ± 0.18	0.623 ± 0.19	0.608 ± 0.052	0.621 ± 0.071	0.639 ± 0.062	**0.667** ± **0.15**
	PPV	0.121 ± 0.063	0.113 ± 0.052	0.106 ± 0.08	0.121 ± 0.089	0.129 ± 0.122	**0.135** ± **0.072**
	NPV	0.941 ± 0.028	0.937 ± 0.033	0.94 ± 0.089	0.942 ± 0.067	0.941 ± 0.05	**0.944** ± **0.035**
Shuffled	AUROC	0.681 ± 0.081	0.635 ± 0.071	0.671 ± 0.113	0.686 ± 0.082	0.662 ± 0.091	**0.698** ± **0.094**
	AUPRC	0.224 ± 0.139	0.169 ± 0.106	0.176 ± 0.11	0.231 ± 0.09	0.174 ± 0.12	**0.251** ± **0.122**
	Sensitivity	0.516 ± 0.151	0.527 ± 0.125	0.549 ± 0.13	0.583 ± 0.083	0.552 ± 0.079	**0.609** ± **0.098**
	Specificity	0.709 ± 0.068	0.652 ± 0.036	0.649 ± 0.072	0.701 ± 0.071	0.678 ± 0.113	**0.712** ± **0.088**
	PPV	0.143 ± 0.08	0.125 ± 0.131	0.121 ± 0.087	0.148 ± 0.094	0.134 ± 0.093	**0.157** ± **0.138**
	NPV	0.947 ± 0.024	0.941 ± 0.03	0.949 ± 0.04	0.951 ± 0.031	0.949 ± 0.041	**0.953** ± **0.018**

The performance is measured as the mean across ten independent runs with different initialisations.

The performance of different methods for the task of mortality prediction on MIMIC-III dataset is documented in Table 4. In the STANDARD scenario, the performance of various FL methods is closely matched, with the exception of the Hypernet-based FL approach. Specifically, the proposed framework outperforms all other methods, achieving an AUROC of 0.831 ± 0.031 and an AUPRC of 0.462 ± 0.04, which are marginally higher than the next best-performing method, AGAT, with an AUROC of 0.83 ± 0.032 and an AUPRC of 0.46 ± 0.021. Notably, while FedAvg achieves strong performance in this scenario, its performance degrades under heterogeneity scenarios. In contrast, the proposed framework demonstrates minimal performance degradation while outperforming comparative baselines, including standalone models, AGAT and LG-FedAvg, convincingly.

**Table 4:** Average performance of the proposed framework and baselines on the MIMIC-III dataset acting as clients in a federated learning setup

Scenario	Metrics	Methods					
		Standalone DNN	FedAvg	Hypernet	LG-FedAvg	AGAT	Proposed
Standard	AUROC	0.811 ± 0.04	0.828 ± 0.032	0.813 ± 0.043	0.821 ± 0.051	0.83 ± 0.032	**0.831** ± **0.031**
	AUPRC	0.431 ± 0.037	**0.46** ± **0.03**	0.434 ± 0.051	0.441 ± 0.028	0.46 ± 0.021	**0.462** ± **0.04**
	Sensitivity	0.682 ± 0.11	**0.729** ± **0.109**	0.693 ± 0.131	0.694 ± 0.091	0.726 ± 0.127	0.724 ± 0.118
	Specificity	0.769 ± 0.057	0.753 ± 0.069	0.749 ± 0.076	0.768 ± 0.058	0.768 ± 0.063	**0.77** ± **0.079**
	PPV	0.282 ± 0.049	**0.285** ± **0.049**	0.273 ± 0.136	0.281 ± 0.042	0.279 ± 0.038	0.284 ± 0.043
	NPV	0.949 ± 0.019	0.956 ± 0.017	0.947 ± 0.041	0.95 ± 0.028	0.952 ± 0.029	**0.958** ± **0.014**
Feature Disparity	AUROC	0.801 ± 0.028	0.77 ± 0.04	0.791 ± 0.032	0.809 ± 0.052	0.812 ± 0.031	**0.821** ± **0.029**
	AUPRC	0.391 ± 0.071	0.365 ± 0.051	0.372 ± 0.052	0.393 ± 0.047	0.406 ± 0.033	**0.412** ± **0.048**
	Sensitivity	0.711 ± 0.131	0.703 ± 0.118	0.709 ± 0.081	0.711 ± 0.112	0.716 ± 0.082	**0.721** ± **0.094**
	Specificity	0.743 ± 0.053	0.749 ± 0.078	0.75 ± 0.058	0.753 ± 0.073	0.757 ± 0.041	**0.761** ± **0.061**
	PPV	0.273 ± 0.034	0.275 ± 0.051	0.271 ± 0.047	0.274 ± 0.059	0.276 ± 0.032	**0.279** ± **0.045**
	NPV	**0.957** ± **0.021**	0.951 ± 0.02	0.942 ± 0.049	0.955 ± 0.047	**0.957** ± **0.057**	0.955 ± 0.051
Shuffled	AUROC	0.811 ± 0.04	0.782 ± 0.047	0.814 ± 0.037	0.815 ± 0.043	0.789 ± 0.035	**0.825** ± **0.028**
	AUPRC	0.431 ± 0.037	0.382 ± 0.048	0.438 ± 0.046	0.438 ± 0.039	0.402 ± 0.047	**0.455** ± **0.028**
	Sensitivity	0.682 ± 0.11	0.709 ± 0.091	0.681 ± 0.11	0.701 ± 0.093	0.689 ± 0.091	**0.716** ± **0.108**
	Specificity	**0.769** ± **0.057**	0.751 ± 0.046	0.759 ± 0.057	0.763 ± 0.061	0.756 ± 0.049	0.768 ± 0.048
	PPV	0.282 ± 0.049	0.276 ± 0.051	0.279 ± 0.072	0.282 ± 0.051	0.278 ± 0.039	**0.283** ± **0.054**
	NPV	0.949 ± 0.019	0.948 ± 0.032	0.949 ± 0.034	0.951 ± 0.039	0.946 ± 0.043	**0.954** ± **0.021**

The performance is measured as the mean across ten independent runs with different initialisations.

**Fig. 5:**
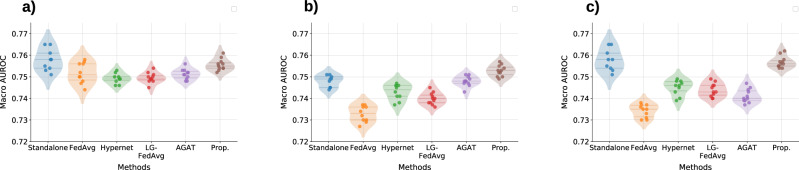
Performance evaluation on MIMIC-III dataset. Assessing the efficacy of the proposed framework on the MIMIC-III dataset for phenotyping across (**a**) standard, (**b**) feature disparity, and (**c**) shuffled data view scenarios, respectively.

Figure 5 documents the performance of different methods for phenotyping on the MIMIC-III dataset in terms of the macro AUROC. In the STANDARD scenario, the proposed framework achieves a score of 0.755 ± 0.003, closely trailing the standalone model, which has an AUROC of 0.757 ± 0.005. While FedAvg, AGAT, Hypernet, and LG-FedAvg also perform well, they exhibit slightly lower AUROCs, with FedAvg at 0.752 ± 0.004, AGAT at 0.751 ± 0.002, Hypernet at 0.748 ± 0.002, and LG-FedAvg at 0.749 ± 0.002. This indicates that the proposed framework is more competitive than the other FL baselines. In the heterogeneity scenarios, the proposed framework outperforms all other FL baselines and exhibits less performance degradation. Moreover, the performance of the standalone models and the proposed framework is comparable, underscoring the effectiveness of the proposed framework in the presence of data view heterogeneity.

### Performance on Graphs data: Modelling CURIAL as Graphs

**Fig. 6:**
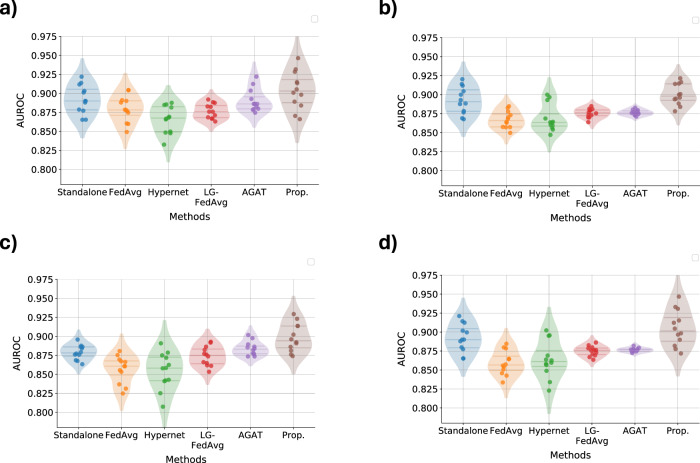
Performance evaluation on graph data type. Performance of the proposed framework on CURIAL datasets being modelled as *graphs* in (**a**) standard, (**b**) feature disparity, (**c**) shuffled, and (**d**) scaled data view scenarios.

At each CURIAL Trust, the tabular data is converted into graphs with patients as nodes (discussed in Methods). These graphs are processed using graph convolutional network (GCN), either as standalone models or in federated baselines. Figure 6 illustrates the performance of each baseline across all experimental scenarios. In STANDARD scenario, the performance of all methods is comparable. However, similar to tabular and time-series datasets, the proposed framework shows better performance on average than the standalone GCN models as well as other federated baselines in presence of data view heterogeneity. Moreover, the proposed framework maintains a consistent average performance despite the presence of data view heterogeneity.

### Do clients use all available features in Feature Disparity scenario?

**Fig. 7:**
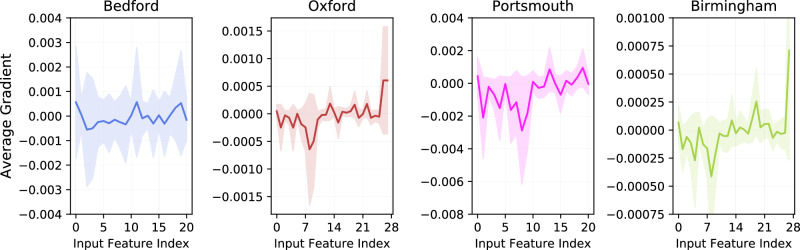
Feature importance in clients’ models trained using the proposed framework. Average gradient of inputs with respect to trained model outputs at each client. The first 21 features are same across all clients. Oxford and Birmingham have additional 7 features.

In FEATURE DISPARITY scenario, at Bedford and Portsmouth CURIAL Trusts, we have 21 blood tests features while the other Trusts (Oxford and Birmingham) contain 7 additional vital signs features including oxygen saturation. To analyse the impact of these additional 7 features, we compute gradient-based input feature importance i.e. gradient of input with respect to model outputs. Figure 7 exhibits the average gradients computed at each CURIAL site. The analysis of this figure highlights that at Oxford and Birmingham, one of the most influential feature (i.e. respiratory rate) is among the 7 additional vital sign features that are not present at Bedford and Portsmouth. This shows that as desired, the proposed framework is able to take into account these additional features rather than just ignoring them.

### Impact of knowledge vector size on the performance

**Fig. 8:**
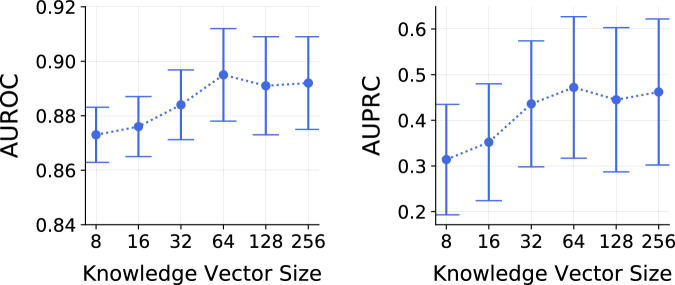
Knowledge vector size impacts the predictive performance. Impact of the knowledge vector size on the performance of the proposed framework on CURIAL datasets in feature disparity scenario.

The dimensionality of the knowledge vector is a critical factor that determines the size of the latent representation obtained after the filtering operation. This representation is then fed into the prediction models within the proposed framework, meaning the dimensionality of the knowledge vector directly influences the complexity of the learned latent representation and, consequently, the performance of the framework. To explore this influence, we varied the dimensionality of the knowledge vector and observed how performance deviates as a function of vector size. Figure 8 illustrates the results of this experiment. Analysis reveals that smaller knowledge vectors, such as those with dimensions of 8 and 16, lead to poorer performance compared to larger vectors with dimensions of 64, 128, and 256. The proposed framework achieves its best performance at a dimensionality of 64, while knowledge vectors with dimensionalities of 128 and 256 also deliver comparable performance. Therefore, selecting the appropriate size for the knowledge vector is crucial. In this work, we treated the dimensionality as a hyperparameter, tuning it to achieve the best validation performance (discussed in Methods).

### Standalone vs federated models with knowledge filtering mechanism

**Fig. 9:**
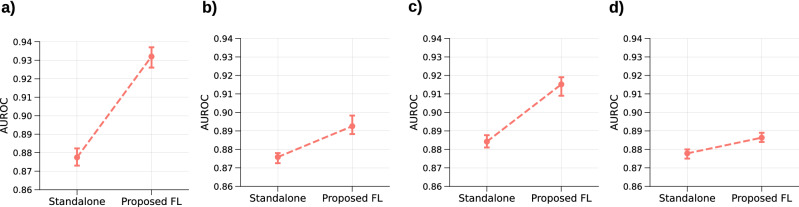
Federated vs standalone training. Performance improvement observed in the proposed FL framework over its standalone variant at (**a**) Bedford, (**b**) Oxford, (**c**) Portsmouth, and (**d**) Birmingham.

To analyse the extent of information sharing in the proposed framework, we implement a standalone version of the *knowledge abstract and filtering* mechanism and compare its performance at each CURIAL site against the proposed framework. In this standalone version, both knowledge vector and filtering module, along with the prediction model, is trained at the client using its local data. The results of this experiment are presented in Fig. 9. The analysis of this figure highlights that federated learning helps in providing better performance than the standalone model. This highlights that information transfer across clients is indeed being facilitated by the proposed framework that would benefit the clients with training data scarcity. Again, this behaviour is evident for *Bedford* that has fewer training examples (Table S2 of supplementary document) and exhibits maximum performance gain of 5.23% over the standalone version.

## Discussion

This paper addressed the challenge of data view heterogeneity in clinical Federated Learning (FL) solutions, which arises from variations in data management practices and the availability of medical services across different institutions serving as federated clients. Substantial preprocessing, often leading to information loss, is necessary to harmonise these data views among clients before they can engage in federated training. The experimental evaluation confirms that information loss incurred during data view alignment, particularly in the FEATURE DISPARITY scenario, can result in a significant performance drop in FedAvg and similar federated solutions. Moreover, advanced FL solutions such as hypernet-based FL, LG-FedAvg, and AGAT, while capable of handling federated training over heterogeneous data views, either fail to address all potential heterogeneity scenarios or struggle to achieve effective information sharing across clients.

These shortcomings, evident in their reduced predictive performance compared to standalone models, highlight the need for a more robust solution. To address this, we introduced a novel FL framework based on a knowledge abstraction and filtering mechanism, which streamlines federated training across heterogeneous data views. This framework minimises the need for labour-intensive data view alignment and improves information transfer among clients. The comprehensive experiments conducted on three extensive healthcare datasets, as well as simulated and real-world scenarios of data view heterogeneity, demonstrate the effectiveness of the proposed framework in achieving strong performance in both homogeneous and heterogeneous data view scenarios across all datasets and data types.

The proposed framework is built on standard FL algorithms, such as FedAvg and FedSGD, and inherits their scalability properties. To evaluate this, we tested the framework with varying numbers of clients, from 4 NHS Trusts in the CURIAL dataset to 50 hospitals in the eICU dataset. The results consistently demonstrate that the framework scales efficiently, maintaining strong performance across different client counts. This highlights its ability to handle a large number of clients, making it highly suitable for real-world FL deployments in healthcare. In terms of computational complexity, the proposed filtering mechanism is lightweight, consisting of only two fully-connected layers (Fig. 2c). When integrated into larger neural networks, the overhead introduced by training the filtering mechanism is negligible, ensuring that the framework remains computationally efficient even with complex models.

The proposed framework offer a significant potential for advancing clinical model development in FL environments, particularly by addressing the challenges posed by data view heterogeneity. This capability not only reduces the additional workload for participating institutions but also encourages greater adoption of federated training, even in scenarios where institutions may have previously hesitated due to data alignment complexities. The framework is particularly effective for clinical applications where data heterogeneity can impede the development of robust models. By enabling seamless learning across institutions with diverse data formats, it supports predictive modelling for a variety of tasks like diagnosis, risk stratification, and personalised treatment without extensive data harmonisation. This adaptability allows institutions to contribute to federated training without disrupting local data management practices. Moreover, this ability to integrate new data sources and clients without significant modifications makes it scalable and suitable for real-world clinical settings. It enhances the development of clinical decision support systems (CDSS), providing clinicians with reliable insights from diverse datasets. By promoting collaboration between healthcare providers, the framework ensures consistent model performance across different institutions and enhances patient care through improved predictive accuracy.

Although Common Data Models (CDMs) aim to harmonise EHR datasets across institutions, their adoption remains limited due to the significant effort required. Our framework focuses instead on enabling deep learning over disparate data views, making it more suitable for federated learning and deep learning scenarios where CDMs may not be feasible.

While this study primarily focuses on addressing data view heterogeneity, it does not explore privacy aspects such as differential privacy, secure aggregation, and secure communication channels. However, since our framework is built on FedAvg, it is compatible with existing privacy-preserving techniques^32–34^, which can be integrated as needed for compliance with data protection regulations in clinical settings. Future work will explore the addition of these privacy measures to enhance patient data security, especially in highly regulated healthcare environments. Another limitation of this study is the lack of evaluation under varying levels of statistical heterogeneity, which is common in healthcare settings. Institutions often differ in terms of patient demographics, disease prevalence, and treatment protocols, leading to diverse data distributions. In future research, we plan to enhance the knowledge abstraction and filtering mechanism by incorporating methods that can address statistical heterogeneity, thereby improving the framework’s robustness and performance across institutions with differing data characteristics. Moreover, this study did not explore the integration of free-text data, a rich source of patient information in healthcare, such as clinical notes. Free-text presents unique challenges due to its unstructured nature, but it has the potential to significantly enhance patient-care models when combined with structured data like clinical features. Future research will aim to develop FL frameworks capable of handling both data modality and data view heterogeneity, integrating free-text data alongside structured EHRs, time-series, and graph data across diverse client environments.

## Methods

The proposed FL framework builds upon the widely used FL algorithm, *FedSGD*, by integrating the novel *knowledge abstraction and filtering* mechanism. This enhancement supports federated training among clients with diverse data views. The knowledge and filtering mechanism projects the input examples at each client, irrespective of their data view, to a fixed length embedding or representation lying in a latent space inherently shared among clients. Predictive modelling is then performed on these “aligned” embeddings rather than the input examples with diverse data structures as illustrated in Fig. 2b. This section first explains the proposed knowledge filtering mechanism and then explains how this mechanism can be exploited in a federated setup to arrive at the proposed FL framework.

### Knowledge filtering mechanism

The proposed filtering mechanism takes two inputs: a *K*-dimensional knowledge vector and a *D*-dimensional sample or feature vector from a client (Fig. 2c). It then generates a *K*-dimensional embedding to be used as input for prediction models. To facilitate this, the proposed mechanism consists of two dense or fully-connected layers with *K* hidden units (*K* is dictated by the size of knowledge vector). One of these layers projects the input sample to *K*-dimensional embedding while the other processes the knowledge vector to a latent *K*-dimensional space. The embeddings generated by these two operations are averaged, followed by a sigmoid activation, to obtain a *K*-dimensional mask which is used to scale or filter the knowledge vector to obtain the desired *K*-dimensional latent or surrogate representation for the input sample. Figure 2c illustrates this whole process. Moreover, following steps describe the knowledge filtering in detail:

- The first linear or dense layer with *K* nodes maps the input example **x** from ${{\mathbb{R}}}^{D}\to {{\mathbb{R}}}^{K}$ : ${{\bf{e}}}_{1}={{\bf{W}}}_{1}^{\top }{\bf{x}}+{{\bf{b}}}_{1}$, where ${{\bf{W}}}_{1}\in {{\mathbb{R}}}^{D\times K}$ and ${{\bf{b}}}_{1}\in {{\mathbb{R}}}^{K}$ are weights and bias of the first linear layer.
- The second linear layer, also having *K* nodes, processes the knowledge vector **h** to obtain ${{\bf{e}}}_{2}\in {{\mathbb{R}}}^{K}$: ${{\bf{e}}}_{2}={{\bf{W}}}_{2}^{\top }{\bf{h}}+{{\bf{b}}}_{2}$, where ${{\bf{W}}}_{2}\in {{\mathbb{R}}}^{K\times K}$ and ${{\bf{b}}}_{2}\in {{\mathbb{R}}}^{K}$ are weights and bias of the second linear layer.
- A filter ${\bf{M}}\in {{\mathbb{R}}}^{K}$ is computed and knowledge vector is filtered to obtain ${\bf{x}}^{\prime} \in {{\mathbb{R}}}^{K}$ as :
  1 $${\bf{M}}=\,\,{{\text{S}}_{\text{IGMOID}}}\,\,\left(\frac{{{\bf{e}}}_{1}+{{\bf{e}}}_{2}}{2}\right),\,\,{\bf{x}}^{\prime} ={\bf{M}}\odot {\bf{h}}.$$
  Here ⊙ is the element-wise multiplication.

This mechanism works by measuring similarity between prominent elements of the input sample and the knowledge vector in the latent space. The average of embeddings basically results in higher magnitude for elements having higher values in embeddings corresponding to both the input sample and the knowledge vector. The following sigmoid activation maps every element of the averaged embedding between 0 and 1 to obtain the filtering mask, where the higher magnitude elements are mapped to larger values around 1 signifying their relevance and vice-versa. This mask is then used to scale the knowledge vector by attenuating its non-relevant elements and emphasising the relevant ones. Hence, the proposed mechanism provides context-based filtering of knowledge vector with context provided by the input samples. Essentially, this knowledge filtering mechanism functions like a simplistic key-value system: the input sample acts as a query to retrieve relevant information from the “knowledge dictionary” (the knowledge vector). By processing the input sample, the mechanism generates keys or a mask to extract the “values” from the knowledge vector.

The proposed filtering mechanism can also be applied in a recursive manner with multiple filtering modules as illustrated in Fig. 2c. The output of the first module is considered as the knowledge vector for the next module while the original input sample remains in the same role as in the first module. This recursive operation is helpful if we are dealing with the complex input representations.

### Proposed FL framework

The proposed framework, similar to *FedSGD* and other FL frameworks, employs a client-server architecture to facilitate distributed model training. In this setup, the server functions as a central entity that defines the global model and oversees its training in a federated manner, without physically accessing any training data. Clients, such as medical institutions and research centres, possess local training data and are willing to participate in the federated training of the global model. As outlined in the Introduction, *FedSGD* and contemporary frameworks operate on an identical blueprint where each training iteration or round consists of the following: (1) The server distributes a copy of the current global model to each client. (2) Clients utilise their local data to compute gradient updates on their respective copies of the global model. (3) Each client sends its computed gradient updates back to the server. (4) The server aggregates these gradient updates and exploits the aggregated update to modify the global model.

The proposed framework also follows the same blueprint while accommodating the knowledge abstraction and filtering mechanism. The overall components and the operations of the proposed framework can be divided among server and clients:

**Server-side processing:** The server finalises the architecture of the global prediction model *f*_*θ*_(), parameterised by *θ*, which processes the *K*-dimensional input samples. Irrespective of the clients’ data views, this model is designed to process *K*-dimensional input samples (either tabular or time-series or graphs). Along with the prediction model, the server also initialises a *K*-dimensional knowledge vector, **h**, with its dimensionality determined by the expected dimensions of the input samples to the global prediction model. During each training round, the server sends a copy of the current global model as well as the knowledge vector to each client, and every *c*-th client provides gradient updates ${\nabla }_{\theta }^{c}$ and ${\nabla }_{{\bf{h}}}^{c}$ for model parameters *θ* and knowledge vector **h** (discussed later). The server aggregates these gradients and update the model and knowledge vector as:

2 $$\theta \leftarrow \theta -\eta \frac{1}{| {\mathcal{C}}| }\sum _{\forall c\in {\mathcal{C}}}{\nabla }_{\theta }^{c},$$

3 $${\bf{h}}\leftarrow {\bf{h}}-\eta \frac{1}{| {\mathcal{C}}| }\sum _{\forall c\in {\mathcal{C}}}{\nabla }_{{\bf{h}}}^{c}.$$

Here ${\mathcal{C}}$ is a set of clients, *η* is the learning rate and ${\nabla }_{\theta }^{c}$ as well as ${\nabla }_{{\bf{h}}}^{c}$ are gradients computed by the *c*-th client. Since the knowledge vector **h** is trained using the gradients obtained from all clients involved in FL, it can be considered as an abstraction or convoluted aggregation of all clients’ information.

**Client-side processing:** Before federated training begins, the participating clients receive detailed information about the global model architecture, including the expected input structure. Every client then finalises its training data, denoted as ${\mathcal{D}}={\{{\bf{x}},y\}}_{j = 1}^{n}$, where **x** is a *D*-dimensional input sample. To handle cases where the client’s data view diverges from the expected input structure, it also initialises a filtering module *F*_*ϕ*_(), parameterised by *ϕ*. This module comprises two fully-connected layers, as illustrated in Fig. 2C. As previously discussed, the filtering module processes an input sample and a knowledge vector to generate a surrogate input representation for the global model.

During a training round, each participating client receives the current global model *f*_*θ*_() and the knowledge vector **h** from the server. The client creates a local copies: ${f}_{{\theta }_{c}}()$ for the global model and **h**_*c*_ for the knowledge vector. The client exploits its filtering mechanism *F*_*ϕ*_() and the knowledge vector **h**_*c*_ to map an input sample (**x**, *y*) to a fixed *K*-dimensional embedding as ${\bf{x}}^{\prime} ={F}_{\phi }({\bf{x}},{{\bf{h}}}_{c})$. This embedding is then fed into the model to obtain prediction $p={f}_{{\theta }_{c}}({\bf{x}}^{\prime} )$. The overall process to obtain prediction for input **x** can be given as: $p={f}_{{\theta }_{c}}({F}_{\phi }({\bf{x}},{{\bf{h}}}_{c}))$. Then, the classification or prediction loss ${\mathcal{L}}$ corresponding to this sample is used to compute gradients and train both prediction model parameters *θ*_*c*_ and filtering module parameters *ϕ*, as well as updating the knowledge vector **h**_*c*_:

4 $$\ell ={\mathcal{L}}({f}_{{\theta }_{c}}({\bf{x}}^{\prime} ),y),\,{\rm{where}}\,\,{\bf{x}}^{\prime} ={F}_{\phi }({\bf{x}},{{\bf{h}}}_{c})$$

5 $${\theta }_{c}={\theta }_{c}-\beta {\nabla }_{{\theta }_{c}}\ell ,\,\,{{\bf{h}}}_{c}={{\bf{h}}}_{c}-\beta {\nabla }_{{{\bf{h}}}_{c}}\ell$$

6 $$\phi =\phi -\beta {\nabla }_{\phi }\ell .$$

Here ${\nabla }_{{\theta }_{c}}\ell$, ${\nabla }_{{{\bf{h}}}_{c}}\ell$ and ∇_*ϕ*_*ℓ* represent the gradient of model parameters *θ*_*c*_, knowledge vector **h**_*c*_ and filtering module parameters *ϕ* with respect to the prediction loss *ℓ*, respectively. The same process is followed for every available input sample. Once this client-side training is complete, the gradients to update global model parameters *θ* and knowledge vector **h** are computed and sent to the server:

7 $${\nabla }_{\theta }^{c}=\theta -{\theta }_{c},\,\,{\nabla }_{{\bf{h}}}^{c}={\bf{h}}-{{\bf{h}}}^{c},$$

where *θ* and **h** are parameter states provided by the server at the beginning of each training round. These gradients are used at the server for model training as defined in Equations 1 and 2. On the other hand, the parameters of trained filtering module, *ϕ*, are stored locally to be utilised in the next training round.

Algorithms 1 and 2 summarises the operations performed at the server and the clients while providing an overall view of the proposed framework.

#### Algorithm 1

**Server-side operations of the proposed** ***knowledge abstraction & filtering*-based federated learning framework**.

// *Run on server*

**Server-Ex**(*n*, *η*):

**Inputs** - Learning rate: *η*, *n*: Training rounds

**Outputs** - Trained model parameters *θ*_*final*_ and trained knowledge vector **h**_*final*_

1: Initialise the global model parameters *θ*_0_ and knowledge vector ${{\bf{h}}}_{0}\in {{\mathbb{R}}}^{K}$

2: **for** *i* ← 1: *n* **do**

3: Select a set of ${\mathcal{C}}$ clients for training

4: **for** **all** *k* ∈ *C* do ⊳ Clients execute in parallel

5: ${\nabla }_{\theta }^{k},{\nabla }_{{\bf{h}}}^{k}\leftarrow$ **Client-Ex**(*θ*_*i*−1_, **h**_*i*−1_) ⊳ Clients return gradients to update *θ* and knowledge vector **h** (Algorithm 2)

6: ${\theta }_{i}\leftarrow {\theta }_{i-1}-\eta \frac{1}{| {\mathcal{C}}| }\sum _{\forall k\in {\mathcal{C}}}{\nabla }_{\theta }^{k}$ ⊳ Aggregating gradients and updating *θ*

7: ${{\bf{h}}}_{i}\leftarrow {{\bf{h}}}_{i-1}-\eta \frac{1}{| {\mathcal{C}}| }\sum _{\forall k\in {\mathcal{C}}}{\nabla }_{{\bf{h}}}^{k}$ ⊳ Aggregating gradients and updating **h**

8: *θ*_*final*_ = *θ*_*i*_, **h**_*final*_ = **h**_*i*_

9: **Return** *θ*_*final*_, **h**_*final*_

#### Algorithm 2

**Client-side operations of the proposed** ***knowledge abstraction & filtering*-based federated learning framework**.

// *Run on clients*

**Client-Ex**(*θ*, **h**):

**Inputs** - Global model parameters: *θ*, Knowledge vector: **h**

**Outputs** - ∇_*θ*_ and ∇_**h**_, gradients to updated *θ* and **h**

1: $${\mathcal{D}}={\{{{\bf{x}}}_{i},{y}_{i}\}}_{i = 1}^{N}$$, Local dataset

2: ${f}_{{\theta }_{c}}()$ and **h**_*c*_, Local model and knowledge vector

3: *F*_*ϕ*_(), filtering module parameterised by *ϕ*

4: *β*, Local learning rate

5: **IF** first training round, initialise *ϕ*. **ELSE** load stored *ϕ*.

6: *θ*_*c*_ = *θ*, **h**_*c*_ = **h** ⊳ Creating copies of *θ* and **h** that are received from the server

7: $${\mathcal{B}}\leftarrow {{\rm{S}}_{\rm{AMPLE}}}\,{{\rm{B}}_{\rm{ATCHES}}}$$ ($${\mathcal{D}}$$) ⊳ $$| {\mathcal{B}}|$$ training batches

8: **for** *j* ← 1: *J* **do** ⊳ Training Epoch

9: **for** **all**$({\bf{x}},y)\in {\mathcal{B}}$ **do**

10: $${\bf{x}}^{\prime} \leftarrow {F}_{\phi }({\bf{x}},{{\bf{h}}}_{c})$$

11: $\ell ={\mathcal{L}}({f}_{{\theta }_{c}}({\bf{x}}^{\prime} ),y)$ ⊳ Compute loss using the local data

12: ${\theta }_{c}={\theta }_{c}-\beta ({\nabla }_{{\theta }_{c}}\ell )$ ⊳ Update local model *θ*_*c*_ using ${\nabla }_{{\theta }_{c}}\ell$, the gradients of loss with respect to *θ*_*c*_

13: ${{\bf{h}}}_{c}={{\bf{h}}}_{c}-\beta ({\nabla }_{{{\bf{h}}}_{c}}\ell )$ ⊳ Update local knowledge vector **h**_*c*_ using ${\nabla }_{{{\bf{h}}}_{c}}\ell$, the gradients of loss with respect to **h**_*c*_

14: *ϕ* = *ϕ* − *β*(∇_*ϕ*_*ℓ*) ⊳ Update filtering module parameters *ϕ* using ∇_*ϕ*_*ℓ*, the gradients of loss with respect to *ϕ*

15: ∇_*θ*_ = *θ* − *θ*_*c*_ ⊳ Computing the gradients for global model parameters *θ*

16: ∇_**h**_ = **h** − **h**_*c*_ ⊳ Computing the gradients for global knowledge vector **h**

17: Locally store *ϕ* for next training round

**Return** ∇_*θ*_, ∇_**h**_

### Importance of knowledge vector based filtering in FL

The proposed filtering mechanism, working in conjunction with the knowledge vector, plays a crucial role in facilitating the learning of a shared representation space among clients. This shared space serves as a surrogate for the input feature space and enables the proposed framework to train a global prediction model, irrespective of differences in clients’ data views. In each training round, the server sends the same knowledge vector to every client. The clients utilise their local filtering mechanisms to compute surrogate embeddings or representations for the input samples that are scaled or filtered version of the knowledge vector. As a result, every client’s surrogate embeddings are conditioned on the same knowledge vector, ensuring minimal deviation from one another. This mechanism minimises distribution shifts among clients and also facilitates information sharing during feature mapping or data view alignment through the knowledge vector. This behaviour is significantly different from contemporary methods such as LG-FedAvg and AGAT, resulting in better federated training and improved performance.

### Additional computational complexity

As previously discussed, the proposed framework differs from the standard FL by incorporating two additional components: a knowledge filtering module and a knowledge vector. In contrast to prediction models, these components entail minimal trainable parameters. Specifically, the knowledge filtering module comprises only two linear layers. Given the utilisation of a large prediction model, the computational burden of implementing knowledge filtering is inconsequential. Regarding client-server communication, the proposed framework necessitates transmitting an extra knowledge vector **h** alongside client gradients. Given that ${\bf{h}}\in {{\mathbb{R}}}^{K}$ is exceedingly small in scale, the impact on client-server communication overhead is infinitesimal.

### Extension to Time-series and Graphs

Till now, we have discussed how feature vectors with different characteristics can be projected onto a *K*-dimensional space defined by **h**. However, the extensions to time-series and graphs are straight-forward. A time-series consists of multiple feature vectors **T** = [**t**_1_, **t**_2_, **t**_3_. . . . **t**_*n*_], and each of these vectors **t**_*i*_ can be mapped to *K*-dimensional space, defined by a knowledge vector **h**, by using the same filtering module. Similarly, in graphs, each node is represented by a feature vector than can be mapped to a fixed *K*-dimensional space defined by **h** using the same filtering module.

### Models and Parameter Setting

The same fully-connected neural network (MLP), featuring a single hidden layer containing 128 nodes, employed in the work of Soltan et al.^12^, is also being employed for processing CURIAL datasets here. For time-series datasets i.e. MIMIC-III and eICU, we have employed LSTM-based binary classifiers^5^. Graph convolutional neural networks (GCN)^35,36^ are employed for handling the graph data type. The detailed architectures of these models are discussed in the supplementary document (Supplementary Note 2).

For all datasets and baselines, we have used a batch-size of 64 examples. The standalone baseline is trained for 300 epochs using Adam optimiser with a learning rate of 0.001. For federated baselines as well as the proposed framework, each framework is trained for *N* = 300 rounds with a global learning rate of *η* = 0.001. At client-side, each framework trains its local model for one training epoch using Adam optimiser with *β* = 0.001 learning rate. The proposed FL framework used 2 filtering modules, one after the other to induce recursive filtering, for knowledge filtering across all datasets. The 64-d knowledge vector is used for CURIAL datasets (both with GCN and DNN). Similarly, 256-d and 512-d knowledge vectors are used for MIMIC-III and eICU datasets, respectively. All these hyperparameters are tuned to obtain the best validation performance.

## Supplementary information

Supplementary Document

## Data Availability

Data from OUH studied here are available from the Infections in Oxfordshire Research Database (https://oxfordbrc.nihr.ac.uk/research-themes/modernising-medical-microbiology-and-big-infection-diagnostics/iord-about/), subject to an application meeting the ethical and governance requirements of the Database. Data from UHB, PUH and BH are available on reasonable request to the respective trusts, subject to HRA requirements. MIMIC-III and eICU datasets are publicly available from https://physionet.org/.

## Code availability

The demo code is available at https://github.com/AnshThakur/Knowledge-Filtering-FL.
