# Publisher Correction: Knowledge abstraction and filtering based federated learning over heterogeneous data views in healthcare

**DOI:** 10.1038/s41746-024-01342-y

**Published:** 2024-11-21

**Authors:** Anshul Thakur, Soheila Molaei, Pafue Christy Nganjimi, Fenglin Liu, Andrew Soltan, Patrick Schwab, Kim Branson, David A. Clifton

**Affiliations:** 1 Department of Engineering Science, University of Oxford, Oxford, UK; 2 Oxford University Hospitals NHS Foundation Trust, Oxford, UK; 3 GlaxoSmithKline, London, UK; 4 Oxford-Suzhou Centre for Advanced Research, Suzhou, China

**Keywords:** Health care, Biomedical engineering

Correction to: *npj Digital Medicine* 10.1038/s41746-024-01272-9, published online 16 October 2024

In this article, author name Fenglin Liu was inadvertently left out. The original article has been corrected.

