# Supplementary material for: Knowledge abstraction and filtering based federated learning over heterogeneous data views in healthcare
Source: NPJ Digit Med. 2024 Oct 16;7:283. doi: 10.1038/s41746-024-01272-9 (PMC11484763; doi:10.1038/s41746-024-01272-9)
Supplement: Supplementary file 1 — Supplementary Document [file 41746_2024_1272_MOESM1_ESM.pdf]

Supplementary Information - Knowledge Abstraction and Filtering based Federated Learning Over Heterogeneous Data Views in Healthcare

Supplementary Note 1- Dataset Details

CURIAL Datasets

The CURIAL database is an anonymized database with United Kingdom National Health Service (NHS) approval via the national oversight/regulatory body, the Health Research Authority (HRA)(CURIAL; NHS HRA IRAS ID: 281832).

Data from Oxford University Hospitals (OUH) studied here are available from the Infections in Oxfordshire Research Database[1], subject to an application meeting the ethical and governance requirements of the Database. Data from University Hospital Birmingham (UHB), Portsmouth University Hospitals (PUH) and Bedford Hospital (BH) are available on reasonable request to the respective trusts, subject to HRA requirements.

Category	Features
Vital Signs	Heart rate, respiratory rate, oxygen saturation, systolic blood pressure, diastolic blood pressure, temperature
Blood Tests	Haemoglobin, haematocrit, mean cell volume, white cell count, neutrophil count, lymphocyte count, monocyte count, eosinophil count, basophil count, platelets
Liver Function Tests & C-reactive protein	Albumin, alkaline phosphatase, alanine aminotransferase, bilirubin, C-reactive protein
Urea & Electrolytes	Sodium, potassium, creatinine, urea, estimated glomerular filtration rate

Supplementary Table 1. Clinical predictors considered for COVID-19 status prediction.

	Bedford	Oxford	Portsmouth	Birmingham
# Examples	1,865	161,955	38,717	95,236
# Positive Examples	210	2,791	2,005	790
# Features	21	28	21	28

Supplementary Table 2. Number of examples at each NHS trust in CURIAL dataset. # Feature represents the dimensionality of data at each client in Feature Disparity scenario.

Tables 1 and 2 document the features and number of examples at each CURIAL site.

MIMIC-III dataset

We processed the MIMIC-III dataset using the publicly available benchmarking code[?]. The duplicate features were removed to obtain 60-d feature vector at each time-step. The number of examples at each simulated clients is documented in Table 3.

List of features in MIMIC-III dataset

1. Capillary refill rate-0.0
2. Capillary refill rate-1.0
3. Diastolic blood pressure
4. Fraction inspired oxygen
5. Glascow coma scale eye opening-2 To Pain
6. Glascow coma scale eye opening-3 To speech
7. Glascow coma scale eye opening-1 No Response
8. Glascow coma scale eye opening-4 Spontaneously
9. Glascow coma scale eye opening-0 None
10. Glascow coma scale motor response-1 No Movement
11. Glascow coma scale motor response-3 Abnormal flexion
12. Glascow coma scale motor response-2 Abnormal extension
13. Glascow coma scale motor response-4 Flex-withdraws
14. Glascow coma scale motor response-5 Localizes Pain

[1] https://oxfordbrc.nihr.ac.uk/research-themes/modernising-medical-microbiology-and-big-infection-diagnostics/infections-in-oxfordshire-research-database-iord/

	Tasks		# Features
	Mortality	Phenotyping	
Client 1	2,113	4,190	60
Client 2	2,113	4,190	55
Client 3	2,113	4,190	48
Client 4	2,113	4,190	55
Client 5	2,113	4,190	60
Client 6	2,113	4,190	48
Client 7	2,113	4,190	57
Client 8	2,113	4,190	60
Client 9	2,113	4,190	48
Client 10	2,122	4,192	55

Supplementary Table 3. Number of examples at each simulated client in MIMIC-III dataset. # Feature represents the dimensionality of data at each client in Feature Disparity scenario.

15. Glascow coma scale motor response-6 Obeys Commands
16. Glascow coma scale total-11
17. Glascow coma scale total-10
18. Glascow coma scale total-13
19. Glascow coma scale total-12
20. Glascow coma scale total-15
21. Glascow coma scale total-14
22. Glascow coma scale total-3
23. Glascow coma scale total-5
24. Glascow coma scale total-4
25. Glascow coma scale total-7
26. Glascow coma scale total-6
27. Glascow coma scale total-9
28. Glascow coma scale total-8
29. Glascow coma scale verbal response-1 No Response
30. Glascow coma scale verbal response-4 Confused
31. Glascow coma scale verbal response-2 Incomprehensible sounds
32. Glascow coma scale verbal response-3 Inappropriate Words
33. Glascow coma scale verbal response-5 Oriented
34. Glucose
35. Heart Rate
36. Height
37. Mean blood pressure
38. Oxygen saturation
39. Respiratory rate
40. Systolic blood pressure
41. Temperature
42. Weight
43. pH
44. mask-Capillary refill rate
45. mask-Diastolic blood pressure
46. mask-Fraction inspired oxygen
47. mask-Glascow coma scale eye opening
48. mask-Glascow coma scale motor response
49. mask-Glascow coma scale total
50. mask-Glascow coma scale verbal response
51. mask-Glucose
52. mask-Heart Rate
53. mask-Height
54. mask-Mean blood pressure
55. mask-Oxygen saturation
56. mask-Respiratory rate
57. mask-Systolic blood pressure
58. mask-Temperature
59. mask-Weight
60. mask-pH

List of 25 patient disorders involved in Phenotyping in MIMIC-III dataset

1. Acute and unspecified renal failure
2. Acute cerebrovascular disease
3. Acute myocardial infarction
4. Cardiac dysrhythmias
5. Chronic kidney disease
6. Chronic obstructive pulmonary disease
7. Complications of surgical/medical care
8. Conduction disorders
9. Congestive heart failure; nonhypertensive
10. Coronary atherosclerosis and related
11. Diabetes mellitus with complications
12. Diabetes mellitus without complication
13. Disorders of lipid metabolism
14. Essential hypertension
15. Fluid and electrolyte disorders
16. Gastrointestinal hemorrhage
17. Hypertension with complications
18. Other liver diseases
19. Other lower respiratory disease
20. Other upper respiratory disease
21. Pleurisy; pneumothorax; pulmonary collapse
22. Pneumonia
23. Respiratory failure; insufficiency; arrest
24. Septicemia (except in labor)
25. Shock

eICU-CRD dataset

We used a pre-processed version of this dataset that is available at https://physionet.org/content/mimic-eicu-fiddle-feature/1.0.0/.

We use this dataset to predict Shock after every 4 hours and hence, each time-series has 4 time-steps. Each time-step is represented by 254-d vectors containing vital signs and demographic features that are listed below:

1. Height: (0, 160.0]
2. Height: (160.0, 167.0]
3. Height: (167.0, 172.7]
4. Height: (172.7, 180.0]
5. Height: (180.0, 612.6]
6. Weight: (0.0, 63.0]
7. Weight: (63.0, 74.0]
8. Weight: (74.0, 85.0]
9. Weight: (85.0, 100.7]
10. Weight: (100.7, 953.0]
11. age: (17.999, 49.0]
12. age: (49.0, 60.0]
13. age: (60.0, 69.0]
14. age: (69.0, 78.0]
15. age: (78.0, 89.0]
16. age: >89
17. Airway type: No Artificial Airway
18. Apache Admission value: Acid-base/electrolyte disturbance
19. Apache Admission value: Angina, unstable
20. Apache Admission value: Bleeding, GI-location unknown
21. Apache Admission value: Bleeding, lower GI
22. Apache Admission value: Bleeding, upper GI
23. Apache Admission value: CHF, congestive heart failure
24. Apache Admission value: CVA, cerebrovascular accident
25. Apache Admission value: Coma/change in level of consciousness
26. Apache Admission value: Diabetic ketoacidosis
27. Apache Admission value: Embolus, pulmonary
28. Apache Admission value: Emphysema/bronchitis
29. Apache Admission value: Endarterectomy, carotid
30. Apache Admission value: Hemorrhage/hematoma, intracranial
31. Apache Admission value: Hypertension, uncontrolled
32. Apache Admission value: Infarction, acute myocardial
33. Apache Admission value: Pneumonia, bacterial
34. Apache Admission value: Renal failure, acute
35. Apache Admission value: Rhythm disturbance (atrial, supraventricular)
36. Apache Admission value: Rhythm disturbance (conduction defect)
37. Apache Admission value: Seizures (primary-no structural brain disease)
38. Apache Admission value: Sepsis, GI
39. Apache Admission value: Sepsis, cutaneous/soft tissue
40. Apache Admission value: Sepsis, pulmonary
41. Apache Admission value: Sepsis, renal/UTI (including bladder)
42. Apache Admission value: Sepsis, unknown
43. Hospital admit offset: (-529268.001, -1790.0]
44. Hospital admit offset: (-1790.0, -389.0]
45. Hospital admit offset: (-389.0, -173.0]
46. Hospital admit offset: (-173.0, -50.0]
47. Hospital admit offset: (-50.0, 118121.0]
48. Hospital admit source: Acute Care/Floor
49. Hospital admit source: Direct Admit
50. Hospital admit source: Emergency Department
51. Hospital admit source: Floor
52. Hospital admit source: Operating Room
53. Hospital admit source: Other Hospital
54. Hospital admit source: Recovery Room
55. Hospital admit source: Step-Down Unit (SDU)
56. Unit admit source: Acute Care/Floor
57. Unit admit source: Direct Admit
58. Unit admit source: Emergency Department
59. Unit admit source: Floor
60. Unit admit source: ICU
61. Unit admit source: ICU to SDU
62. Unit admit source: Operating Room
63. Unit admit source: Other Hospital
64. Unit admit source: Other ICU
65. Unit admit source: PACU
66. Unit admit source: Recovery Room
67. Unit admit source: Step-Down Unit (SDU)',
68. Unit stay type: admit
69. Unit stay type: readmit
70. Unit stay type: stepdown/other
71. Unit stay type: transfer
72. Unit type: CCU-CTICU
73. Unit type: CSICU
74. Unit type: CTICU
75. Unit type: Cardiac ICU
76. Unit type: MICU
77. Unit type: Med-Surg ICU
78. Unit type: Neuro ICU
79. Heart Rate mask
80. Non-Invasive BP Diastolic mask
81. Non-Invasive BP Systolic mask
82. O_2 Saturation mask
83. Respiratory Rate mask
84. CVP (-34.001, 5.0]
85. CVP (5.0, 8.0]
86. CVP (8.0, 11.0]
87. CVP (11.0, 15.0]
88. CVP (15.0, 396.0]
89. Invasive BP Diastolic (<48.0)
90. Invasive BP Diastolic (48.0, 56.0]
91. Invasive BP Diastolic (56.0, 62.0]
92. Invasive BP Diastolic (62.0, 71.0]
93. Invasive BP Diastolic (>71.0)
94. Invasive BP Mean (-50.001, 68.0]
95. Invasive BP Mean (68.0, 76.0]
96. Invasive BP Mean (76.0, 84.0]
97. Invasive BP Mean (84.0, 94.0]
98. Invasive BP Mean (>94.0)
99. Invasive BP Systolic (<104.0)
100. Invasive BP Systolic (104.0, 118.0]
101. Invasive BP Systolic (118.0, 131.0]
102. Invasive BP Systolic (131.0, 146.0]
103. Invasive BP Systolic (>146.0)
104. Non-Invasive BP Mean (<67.0)
105. Non-Invasive BP Mean (67.0, 76.0]
106. Non-Invasive BP Mean (76.0, 84.0]
107. Non-Invasive BP Mean (84.0, 95.0]
108. Non-Invasive BP Mean (>95.0)
109. O_2 Admin Device: BiPAP
110. O_2 Admin Device: BiPAP/CPAP
111. O_2 Admin Device: NC
112. O_2 Admin Device: RA
113. O_2 Admin Device: nasal cannula
114. O_2 Admin Device: nc
115. O_2 Admin Device: non-rebreather
116. O_2 Admin Device: other
117. O_2 Admin Device: ra
118. O_2 Admin Device: room air
119. O_2 Admin Device: trach collar
120. O_2 Admin Device: ventilator
121. O_2 Admin Device: venturi mask
122. O_2 L%: (<2.0)
123. O_2 L%: (2.0, 3.0]
124. O_2 L%: (3.0, 6.0]
125. O_2 L%: >6.0
126. Temperature (C): (<36.4)
127. Temperature (C): (36.4, 36.7]
128. Temperature (C): (36.7, 36.9]
129. Temperature (C): (36.9, 37.2]
130. Temperature (C): (>37.2)
131. Temperature (F): (<97.5)
132. Temperature (F): (97.5, 98.1]
133. Temperature (F): (98.1, 98.4]
134. Temperature (F): (98.4, 99.0]
135. Temperature (F): (>99.0)
136. Temperature Location: (-0.001, 1.0]
137. Temperature Location: (1.0, 4.0]
138. Temperature Location: TA
139. Temperature Location: AXILLARY
140. Temperature Location: BLADDER
141. Temperature Location: Core urinary catheter
142. Temperature Location: Forehead
143. Temperature Location: Oral
144. Temperature Location: PA CATHETER
145. Temperature Location: Rectal
146. Temperature Location: Skin Sensor
147. Temperature Location: TEMPORAL
148. Temperature Location: TEMPORAL ARTERY
149. Temperature Location: TYMPANIC
150. Temperature Location: Temporal Artery Scan
151. Temperature Location: Temporal scan
152. Temperature Location: core
153. Temperature Location: undocumented
154. Non-Invasive BP Diastolic delta time: (-0.001, 1.0]
155. Non-Invasive BP Diastolic delta time: (-0.001, 1.0]
156. Heart Rate: (-0.001, 68.0]
157. Heart Rate: (68.0, 78.0]
158. Heart Rate: (78.0, 88.0]
159. Heart Rate: (88.0, 100.0]
160. Heart Rate: (100.0, 300.0]
161. Non-Invasive BP Diastolic: (-0.001, 54.0]
162. Non-Invasive BP Diastolic: (54.0, 61.0]
163. Non-Invasive BP Diastolic: (61.0, 69.0]
164. Non-Invasive BP Diastolic: (69.0, 79.0]
165. Non-Invasive BP Diastolic: (79.0, 866.0]
166. Non-Invasive BP Systolic: (-0.001, 102.0]
167. Non-Invasive BP Diastolic: (102.0, 114.0]
168. Non-Invasive BP Diastolic: (114.0, 127.0]
169. Non-Invasive BP Systolic: (127.0, 142.0]
170. Non-Invasive BP Diastolic: (142.0, 12065.0]
171. O_2 Saturation: (-0.001, 95.0]
172. O_2 Saturation: (95.0, 96.0]

173. O_2 Saturation: (96.0, 98.0]
174. O_2 Saturation: (98.0, 99.0]
175. O_2 Saturation: (99.0, 999.0]
176. Respiratory Rate: (-0.001, 15.0]
177. Respiratory Rate: (15.0, 17.0]
178. Respiratory Rate: (17.0, 20.0]
179. Respiratory Rate: (20.0, 24.0]
180. Respiratory Rate: (24.0, 912.0]
181. Heart Rate min: (-0.001, 67.0]
182. Heart Rate min: (67.0, 77.0]
183. Heart Rate min: (77.0, 87.0]
184. Heart Rate min: (87.0, 99.0]
185. Heart Rate min: (99.0, 293.0]
186. Heart Rate max: (-0.001, 69.0]
187. Heart Rate max: (69.0, 79.0]
188. Heart Rate max: (79.0, 88.0]
189. Heart Rate max: (88.0, 101.0]
190. Heart Rate max: (101.0, 959.0]
191. Heart Rate mean: (-0.001, 68.0]
192. Heart Rate mean: (68.0, 78.0]
193. Heart Rate mean: (78.0, 87.5]
194. Heart Rate mean: (87.5, 100.0]
195. Heart Rate mean: (100.0, 527.0]
196. Non-Invasive BP Diastolic min: (-0.001, 52.0]
197. Non-Invasive BP Diastolic min: (52.0, 60.0]
198. Non-Invasive BP Diastolic min: (60.0, 68.0]
199. Non-Invasive BP Diastolic min: (68.0, 78.0]
200. Non-Invasive BP Diastolic min: (78.0, 777.0]
201. Non-Invasive BP Diastolic max: (-0.001, 55.0]
202. Non-Invasive BP Diastolic max: (55.0, 63.0]
203. Non-Invasive BP Diastolic max: (63.0, 70.0]
204. Non-Invasive BP Diastolic max: (70.0, 80.0]
205. Non-Invasive BP Diastolic max: (80.0, 6078.0]
206. Non-Invasive BP Diastolic mean: (-0.001, 54.0]
207. Non-Invasive BP Diastolic mean: (54.0, 61.25]
208. Non-Invasive BP Diastolic mean: (61.25, 69.0]
209. Non-Invasive BP Diastolic mean: (69.0, 79.0]
210. Non-Invasive BP Diastolic mean: (79.0, 1578.5]
211. Non-Invasive BP Systolic min: (-0.001, 100.0]
212. Non-Invasive BP Systolic min: (100.0, 113.0]
213. Non-Invasive BP Systolic min:(113.0, 125.0]
214. Non-Invasive BP Systolic min: (125.0, 141.0]
215. Non-Invasive BP Systolic min: (141.0, 12065.0]
216. Non-Invasive BP Systolic max: (-0.001, 104.0]
217. Non-Invasive BP Systolic max: (104.0, 116.0]
218. Non-Invasive BP Systolic max: (116.0, 128.0]
219. Non-Invasive BP Systolic max: (128.0, 144.0]
220. Non-Invasive BP Systolic max: (144.0, 12065.0]
221. Non-Invasive BP Systolic mean: (-0.001, 102.0]
222. Non-Invasive BP Systolic mean: (102.0, 114.0]
223. Non-Invasive BP Systolic mean: (114.0, 126.5]
224. Non-Invasive BP Systolic mean: (126.5, 142.0]
225. Non-Invasive BP Systolic mean: (142.0, 12065.0]
226. O_2 Saturation min: (-0.001, 94.0]
227. O_2 Saturation min: (94.0, 96.0]
228. O_2 Saturation min: (96.0, 98.0]
229. O_2 Saturation min: (98.0, 99.0]
230. O_2 Saturation min: (99.0, 999.0]
231. O_2 Saturation max: (-0.001, 95.0]
232. O_2 Saturation max: (95.0, 97.0]
233. O_2 Saturation max: (97.0, 98.0]
234. O_2 Saturation max: (98.0, 100.0]
235. O_2 Saturation mean: (-0.001, 95.0]
236. O_2 Saturation mean: (95.0, 96.0]
237. O_2 Saturation mean: (96.0, 98.0]
238. O_2 Saturation mean: (98.0, 99.0]
239. O_2 Saturation mean: (99.0, 999.0]
240. Respiratory Rate min: (-0.001, 14.0]
241. Respiratory Rate min: (14.0, 17.0]
242. Respiratory Rate min: (17.0, 19.0]
243. Respiratory Rate min: (19.0, 23.0]
244. Respiratory Rate min: (23.0, 912.0]
245. Respiratory Rate max: (-0.001, 15.0]
246. Respiratory Rate max: (15.0, 18.0]
247. Respiratory Rate max: (18.0, 20.0]
248. Respiratory Rate max: (20.0, 24.0]
249. Respiratory Rate max: (24.0, 2122.0]
250. Respiratory Rate mean: (-0.001, 15.0]
251. Respiratory Rate mean: (15.0, 17.667]
252. Respiratory Rate mean: (17.667, 20.0]
253. Respiratory Rate mean: (20.0, 23.25]
254. Respiratory Rate mean: (23.25, 912.0]

The number of examples at each of 50 hospital sites (clients) are documented in Table 4.

Hospital ID	# Examples	# Features
73	6230	100
264	4587	100
167	4409	100
338	3620	216
208	3401	216
243	3282	254
443	3183	254
458	3180	254
420	3157	254
119	3114	254
176	3114	100
300	3073	100
122	2862	100
188	2711	254
252	2709	254
411	2703	216
413	2293	216
165	2152	254
171	2118	216
281	1946	216
148	1914	216
283	1898	254
110	1869	216
449	1817	216
394	1740	100
331	1723	254
440	1700	216
417	1679	100
416	1658	254
307	1618	254
365	1564	254
197	1558	216
345	1478	100
157	1474	254
435	1426	254
63	1409	254
400	1401	216
141	1390	100
277	1330	254
248	1253	254
183	1249	254
142	1237	216
217	1225	100
382	1223	254
444	1197	254
146	1192	254
318	1191	216
154	1182	100
79	1149	254
310	1135	254

Supplementary Table 4. Number of examples at each simulated client in eICU-CRD dataset. # Feature represents the dimensionality of data at each client in Feature Disparity scenario.

	CLIENT 1	CLIENT 2	CLIENT 3	CLIENT 4	CLIENT 5
# EXAMPLES	800	800	800	800	800
# POSITIVE EXAMPLES	105	102	129	106	112
# FEATURES	25	44	32	44	40

Supplementary Table 5. Number of examples at each simulated client in PhysioNet dataset. # FEATURE represents the dimensionality of data at each client in FEATURE DISPARITY scenario.

PhysioNet 2012 dataset

This document also presents additional results on PhysioNet 2012 dataset.

LIST OF FEATURES IN PHYSIONET 2012 DATASET

1. Alkaline phosphatase
2. Alanine transaminase
3. Aspartate transaminase
4. Albumin
5. Blood urea nitrogen
6. Bilirubin
7. Cholesterol
8. Creatinine
9. Invasive diastolic arterial blood pressure
10. Fractional inspired oxygen
11. Glasgow Comma Score
12. Glucose
13. Serum bicarbonate
14. Hematocrit
15. Heart rate
16. Height
17. ICU Type - Coronary Care Unit
18. ICU Type - Cardiac Surgery Recovery Unit
19. ICU Type - Medical ICU
20. ICU Type - Surgical ICU
21. Serum potassium
22. Lactate
23. Invasive mean arterial blood pressure
24. Mechanical ventilation respiration
25. Serum magnesium
26. Non-invasive diastolic arterial blood pressure
27. Non-invasive mean arterial blood pressure
28. Non-invasive systolic arterial blood pressur
29. Serum sodium
30. Partial pressure of arterial CO_2
31. Partial pressure of arterial O_2
32. Platelets
33. Respiration rate
34. SAPS-I score
35. SOFA score
36. O_2 saturation in hemoglobin
37. Invasive systolic arterial blood pressure
38. Temperature
39. Troponin-I
40. Troponin-T
41. Urine output
42. White blood cell count
43. Weight
44. Arterial pH

The number of examples at each simulated client is presented in Table 5.

Supplementary Note 2 - Model Architectures

DNN For CURIAL:

DENSE LAYER WITH 128 NODES → RELU ACTIVATION → DROPOUT WITH 0.25 RATE → DENSE LAYER WITH 1 NODE → SIGMOID ACTIVATION

LSTM For PhysioNet, MIMIC and eICU:

LSTM WITH 128 NODES → RELU ACTIVATION → DROPOUT WITH 0.25 RATE → DENSE LAYER WITH C NODES → SIGMOID ACTIVATION

C is set to 1 and 25 for prediction and phenotyping, respectively.

Graph Convolutional Network (GCN):

GCNCONV (128 NODES) → RELU ACTIVATION → DROPOUT WITH 0.25 RATE → DENSE LAYER WITH C NODES → SIGMOID ACTIVATION

Here GCNCONV stands for graph convolutional layer [**?**].

Hypernetwork architectures used in baselines

The code provided at https://github.com/AvivSham/pFedHN has been adapted to design hypernetworks catering to models discussed above.

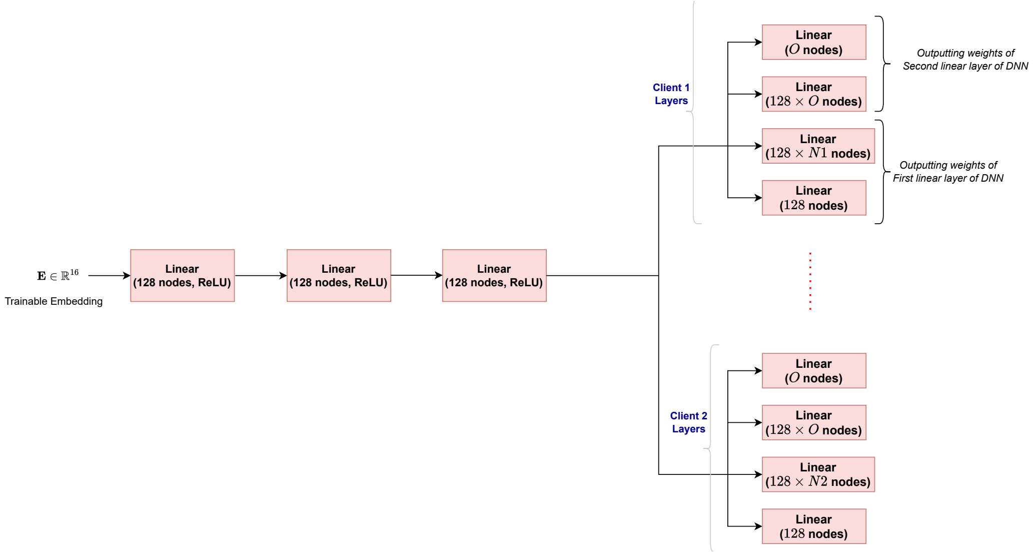

Supplementary Figure 1. Hypernetwork architecture used for the CURIAL clients. For each client, there is a separate head outputting weights for the DNN architecture discussed above. Here $O = 1$ is the number of output classes, $N1$ and $N2$ are the number of input features at each client.

HYPERNETWORKS: The hypernet architectures used for CURIAL and time-series datasets are illustrated in Figures 1 and 2, respectively. The spectral normalisation is applied on initialised weights of hypernetworks.
Similar to other FL baselines, the hypernetwork at server is updated using a learning of 0.001. Whereas, each local model at client is trained using Adam optimiser with a learning rate of 0.001.

Supplementary Note 3 - Additional results on PhysioNet dataset

We also conducted the performance evaluation of the proposed framework and other baselines on PhysioNet 2012 challenge dataset. This is a time-series dataset with 48 time-steps and 44 features at each step. The task is to perform mortality prediction based on first 48 hours of ICU stay. To simulate federated settings, we divided this dataset among 5 simulated clients. The feature disparity scenario is simulated by randomly drooping some features at certain clients. We used LSTM with 256 recurrent units as the prediction model for this task. The knowledge vector of 256 dimensions is used in the proposed framework. Parameters setting used for MIMIC-III is also used here and is found to be optimal. The results of this experiment are documented in 3. The analysis of this figure again shows than the proposed framework performs well in presence of data view heterogeneity.

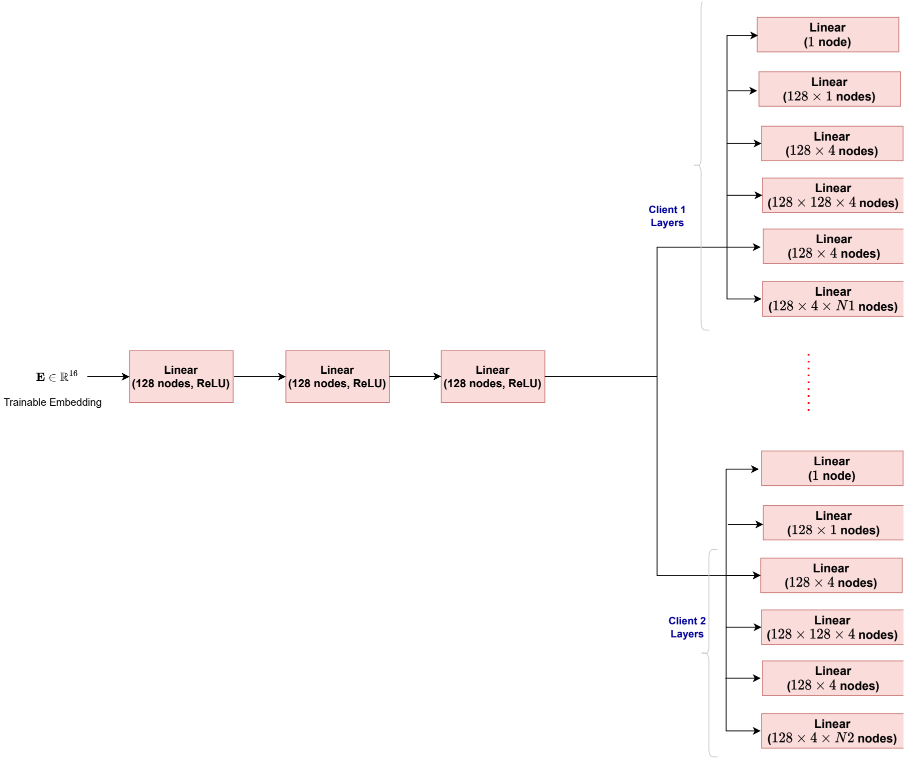

Supplementary Figure 2. Hypernetwork architecture used for the LSTM clients' models. For each client, there is a separate head outputting weights for the LSTM architecture discussed above. Here 1 is the number of output classes, $N1$ and $N2$ are the number of input features at each client.

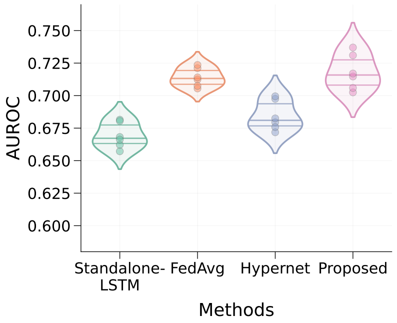

(a) STANDARD

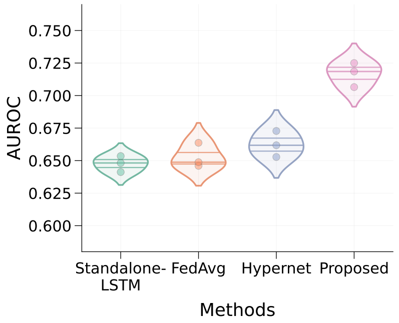

(b) FEATURE DISPARITY

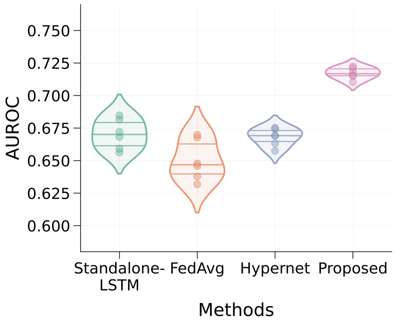

(c) SHUFFLED

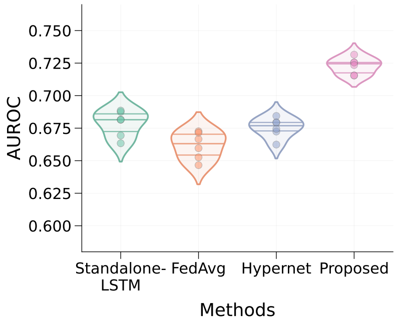

(d) SCALED

Supplementary Figure 3. Performance evaluation on PhysioNet 2012 Challenge dataset. Performance of the proposed framework in a) standard, b) feature disparity, c) shuffled data views, and d) scaled scenarios.
